# Dramatic Number Variation of *R* Genes in Solanaceae Species Accounted for by a Few *R* Gene Subfamilies

**DOI:** 10.1371/journal.pone.0148708

**Published:** 2016-02-05

**Authors:** Chunhua Wei, Jiongjiong Chen, Hanhui Kuang

**Affiliations:** 1 Key Laboratory of Horticultural Plant Biology, Ministry of Education, and Key Laboratory of Horticultural Crop Biology and Genetic improvement (Central Region), MOA, College of Horticulture and Forestry Sciences, Huazhong Agricultural University, Wuhan, P.R. China, 430070; 2 College of Horticulture, Northwest A&F University, Yangling, Shanxi, China, 712100; National Taiwan University, TAIWAN

## Abstract

Most disease resistance genes encode nucleotide-binding-site (NBS) and leucine-rich-repeat (LRR) domains, and the NBS-LRR encoding genes are often referred to as *R* genes. Using newly developed approach, 478, 485, 1,194, 1,665, 2,042 and 374 *R* genes were identified from the genomes of tomato Heinz1706, wild tomato LA716, potato DM1-3, pepper Zunla-1 and wild pepper Chiltepin and tobacco TN90, respectively. The majority of *R* genes from Solanaceae were grouped into 87 subfamilies, including 16 TIR-NBS-LRR (TNL) and 71 non-TNL subfamilies. Each subfamily was annotated manually, including identification of intron/exon structure and intron phase. Interestingly, TNL subfamilies have similar intron phase patterns, while the non-TNL subfamilies have diverse intron phase due to frequent gain of introns. Prevalent presence/absence polymorphic *R* gene loci were found among Solanaceae species, and an integrated map with 427 *R* loci was constructed. The pepper genome (2,042 in Chiltepin) has at least four times of *R* genes as in tomato (478 in Heinz1706). The high number of *R* genes in pepper genome is due to the amplification of *R* genes in a few subfamilies, such as the *Rpi-blb2* and *BS2* subfamilies. The mechanism underlying the variation of *R* gene number among different plant genomes is discussed.

## Background

Plants harbor a variety of disease resistance genes to protect themselves from their natural enemies, such as pests, viruses and fungi. Up to now, more than 140 resistance genes have been cloned and well characterized from flowering plants, of which approximately 80% encode nucleotide-binding-site (NBS) and leucine-rich-repeat (LRR) domains [1–3]. The NBS-LRR encoding genes belong to a large gene family, with hundreds of copies in a genome [4–6]. Based on their N-terminal structures, these R proteins can be further divided into two subclasses: TIR-NBS-LRR (TNL) that possesses a domain homologous to the Toll and interleukin-1 receptor (TIR), and non-TNL. Most non-TNL R proteins have a coiled-coil (CC) structure at the N terminal and are often called CC-NBS-LRR (CNL) R proteins [7,8]. For convenience, all NBS-LRR encoding genes and their truncated close homologs are referred to *R* genes hereafter in this manuscript.

*R* genes have been identified from genomes of several plant species. For instance, 159 and 185 *R* genes were identified from the genomes of *Arabidopsis thaliana* and *A*. *lyrata*, respectively [9]; 623 and 725 from rice cultivars Nipponbare and 93–11, respectively [5]; 292 from Brachypodium [10]; 459 and 330 from woody species grape and poplar, respectively [11]; 355 from cotton [12]; 1,015 from apple [13]; 571, 289, 337 and 465 from four legume species [3]; 755, 394 and 684 from potato (*Solanum tuberosum*), tomato (*S*. *lycopersicum*) and pepper (*Capsicum annuum*), respectively [14,15]. Some species contain relative few *R* genes in genomes, for example, only 54 in the papaya genome [16] and 55–75 from an individual Cucurbitaceae species [17].

The majority of *R* genes tend to be physically clustered in plant genomes, forming gene clusters (also called *R* loci or multiple-copy *R* loci) [18]. For example, 109 of the 149 NBS-LRR genes in Arabidopsis were organized in clusters [8,18]; 119 multiple-copy loci were detected in the genome of rice cultivar Nipponbare, composing the majority (74.3%) of *R* genes (623) annotated in rice [5]; similarly, 577 out of 755 *R* genes in potato were located within a total of 92 clusters [14]. Gene duplication was considered to have played an important role in the expansion of an *R* gene family [3,10,13].

Comparative analysis revealed that presence/absence (P/A) polymorphism is prevalent between species [5,9,19,20]. An integrated map of *R* gene loci for a plant family will be helpful to understand the distribution of P/A polymorphism as well as their evolutionary mechanism, and it is also useful for future mapping and cloning of *R* genes [3,5]. *R* gene family represents the most divergent gene family in plant genome, with considerable copy number variation, P/A polymorphism as well as sequence variation caused by various evolutionary mechanisms [5,18,20,21].

*R* genes have been identified and compared between two closely related Solanaceae species, tomato and potato [14,22–25]. The genomes of some important Solanaceae species have been sequenced and released recently, but comparison of *R* genes and their evolution in Solanaceae species remain to be a comprehensively investigated [15,26–32].

In this study, we re-identified *R* genes from Solanaceae species and hundreds of additional *R* genes were obtained. Their distribution, organization, classification, and P/A polymorphism were analyzed. All *R* gene subfamilies were manually annotated, providing reference gene model for future studies of *R* genes in Solanaceae species. Using tomato Heinz1706 genome as a reference, an integrated *R* gene map with 427 *R* loci was constructed for Solanaceae, which may facilitate future *R* gene cloning from Solanaceae species. The mechanism for copy number variation of *R* genes among Solanaceae species was studied in detail.

## Results

### Identification of *R* Genes in Solanaceae Species

Using a BLASTN method (details in MM section) [5], 465, 485, 1,185, 1,665, 2,042 and 374 *R* genes were identified from the genomes of tomato cultivar Heinz1706, wild tomato LA716 (*S*. *pennellii*), potato DM1-3, pepper cultivar Zunla-1, wild pepper Chiltepin (*C*. *annuum* var. *glabriusculum*) and tobacco (*Nicotiana tabacum*) TN90, respectively (Table 1, S1 and S2 Datas). Compared with previous studies [14,15], 121 and 450 additional *R* genes were obtained from the genomes of tomato Heinz1706 and potato DM1-3 (S1 Table). Most (> 80%) of the newly identified genes do not have the NBS encoding sequences, but are highly similar to at least one NBS-LRR encoding genes, and therefore are partial *R* genes. Meanwhile, 13 (from tomato Heinz1706) and 9 *R* homologs (from potato DM1-3) identified by previous studies were missed using our method and were also included for further analysis (S1 and S2 Tables). Consequently, a total of 478 and 1,194 *R* genes from Heinz1706 and DM1-3 were used for further analysis (S1 and S2 Tables).

**Table 1:** The number of *R* genes and their distribution on different chromosomes of Solanaceae species.

	Tomato (Heinz1706)	Tomato (LA716)	Potato (DM1-3)	Pepper (Zunla-1)	Pepper (Chiltepin)	Tobacco (TN90)
**Chr00**	7	12	226	636	1050	/
**Chr01**	31	38	68	104	81	/
**Chr02**	30	27	33	27	49	/
**Chr03**	11	14	15	116	115	/
**Chr04**	81	65	159	30	44	/
**Chr05**	61	58	88	141	107	/
**Chr06**	27	40	125	91	64	/
**Chr07**	17	17	21	51	57	/
**Chr08**	21	19	81	31	58	/
**Chr09**	49	55	101	177	154	/
**Chr10**	42	54	80	95	97	/
**Chr11**	54	53	124	92	97	/
**Chr12**	34	33	64	74	69	/
**Total number**	465	485	1185	1665	2042	374

Chr00 represents unanchored supper-scaffolds.

The number of *R* genes in the genome of pepper (Chiltepin, in particular) is considerably more than that in most other plant genomes sequenced so far (S3 Table). On the other hand, some other Solanaceae species, such as tobacco and tomato, have only a moderate number of *R* genes (Table 1). The *R* gene copy number is inconsistent with the number of predicted genes or genome sizes among Solanaceae species. For example, the tetraploid tobacco has the largest genome and the largest number of predicted genes, but has low *R* gene number. The large variation of *R* gene copy number among different Solanaceae genomes is in striking contrast to the similar number of *R* genes in different Cucurbitaceae species [17].

### Classification of 87 *R* Gene Subfamilies in Solanaceae

To facilitate *R* gene identification and annotation in future studies, all *R* genes identified from Solanaceae species were classified into subfamilies. Using BLASTN method (E-value < 1e^-10^), the majority (more than 92%) of *R* genes in Solanaceae can be divided into 87 subfamilies, while the remaining were partial and could not be grouped into any subfamilies. A subfamily is named after a well characterized or a randomly chosen full-length gene in a group, such as subfamily *ZL-0810*. Fourteen of the 87 subfamilies have at least one *R* gene well characterized in previous studies. For the remaining 73 subfamilies, one full-length sequence was chosen to annotate manually (see MM section). They (one for each of the 87 subfamilies) are referred to as ref-genes hereafter. The 87 subfamilies include 16 TNL and 71 nTNL types (Table 2 and S4 Table). The classification of all *R* genes and their detailed annotation will help future cloning and sequence analysis of *R* genes in Solanaceae.

**Table 2:** *R* gene subfamilies in Solanaceae species.

	Tomato (Heinz1706)	Tomato (LA716)	Potato (DM1-3)	Pepper (Zunla-1)	Pepper (Chiltepin)	Tobacco (TN90)	Ref-genes
**Total no. of Ref-genes**	81	79	83	80	83	54	87
**No. of *R* genes not homologous to Ref-genes**	10	9	28	61	94	28	/
**No. of *R* genes homologous to Ref-genes**	468	476	1166	1604	1948	346	/
**No. of TNL genes**	98	97	250	177	204	69	16
**No.of nTNL genes**	370	379	916	1427	1744	277	71
**No. of subfamily with one homolog**	38	31	20	16	16	13	/
**No. of subfamily with ten or more homologs**	14	15	23	31	30	14	/
**Average No. of *R* genes in a subfamily**	5.8	6.0	14.1	20.1	23.5	6.4	/

### The Evolution of *R* gene Subfamilies

**Fig 1:**
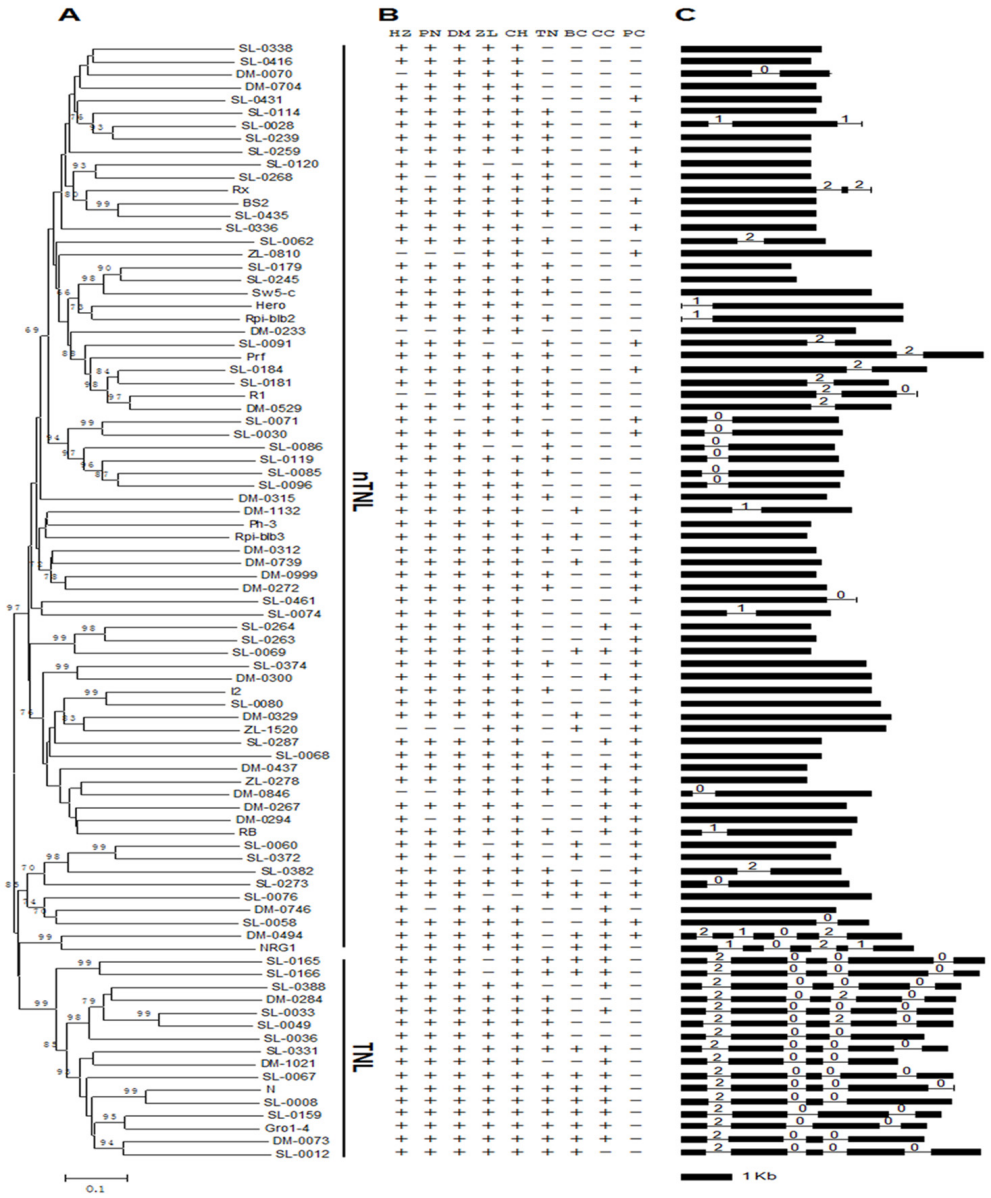
(A). Distance tree for the 87 ref-genes in Solanaceae. Amino acid sequences of NBS region of the 87 ref-genes were used to construct the tree. The ref-genes from tomato Heinz1706, potato DM1-3 and pepper Zunla-1 were named as *HZ-*, *DM-* and *ZL-* followed by a number, respectively. Numbers on nodes are bootstrap values, and values <65 are not shown. (B). P/A polymorphisms of the 87 subfamilies in four plant families (Solanaceae, Brassicaceae, Cucurbitaceae and Poaceae). HZ, PN, DM, ZL, CH, TN, BC, CC and PC on the top line represent tomato Heinz1706, tomato LA716, potato DM1-3, pepper Zunla-1, pepper Chiltepin, tobacco TN90, Brassicaceae, Cucurbitaceae and Poaceae, respectively. The mark “+” represent presence of the subfamily. (C). Gene models for the 87 ref-genes in Solanaceae. Black boxes represent exons, while lines linking boxes represent introns. The number indicates intron phase: 0 = intron phase 0; 1 = intron phase 1; 2 = intron phase 2. The bar represents the scale of exon, while introns are not drawn to scale.

A distance tree was constructed using predicted amino acid sequences of the NBS domain of the 87 subfamilies. As expected, the 16 TNL subfamilies and the 71 nTNL subfamilies are grouped into two major lineages, respectively (Fig 1A).

To study the origin and evolution of the 87 *R* subfamilies from Solanaceae, they were compared with the *R* genes from Brassicaceae (*A*. *thaliana*), Poaceae (rice, maize, sorghum and *Brachypodium distachyon*) and Cucurbitaceae species (cucumber, melon and watermelon) using TBLASTX [5,9,17]. If a Solanaceae *R* gene subfamily is present in a genome, “+” is marked in corresponding position in Fig 1B. As expected, the 16 TNL subfamilies are not present in Poaceae. Interestingly, most of the 71 nTNL subfamilies are found in Poaceae (monocot) but more than half of them are absent in Cucurbitaceae and Arabidopsis (dicot), suggesting frequent loss of *R* subfamilies in these two plant families (Fig 1B).

The P/A polymorphism of *R* subfamilies also occurs among different Solanaceae species (Fig 1A and 1B). For instance, the *ZL-0810* and *ZL-1520* subfamilies are present in pepper but absent in Solanum (potato and tomato); the *DM-0233*, *DM-0846* and *R1* subfamilies are present in potato but lost in tomato; the *SL-0076* subfamily is lost in pepper; the *SL-0165* and *SL-0166* subfamilies are absent in pepper cultivar Zunla-1. The P/A polymorphism between different Solanaceae species showed that *R* gene subfamilies might be lost during a short evolutionary period.

### Intron Feature Variations and Intron Gain/Loss in TNL and nTNL Subfamilies

The intron-exon boundaries and intron phase of the 87 ref-genes of Solanaceae were studied and compared. Since members in each subfamily are close homologs, it was assumed that the gene structure of each ref-gene represents that of all genes in corresponding subfamily. Surprisingly, the average number of exons for each subfamily varies dramatically between the TNL and nTNL groups, though the two groups have similar gene length. Of the 71 nTNL subfamilies, 42 have only a single exon, 24 have two exons and 5 have more than two exons, while all 16 TNL subfamilies have 4 or 5 exons (Fig 1C and S4 Table).

The distance tree and the gene structure (intron position and intron phase) suggest that the common ancestor of the nTNL lineage had no introns since none of the introns were present in two distantly related nTNL subfamilies (Fig 1C). Most (42) nTNL subfamilies have no intron, and these subfamilies with no intron spread all over the distance tree. For the 29 subfamilies with intron, they are always closely related if they have the same intron feature. For example, subfamilies *SL-0030*, *SL-0071*, *SL-0086*, *SL-0085*, *SL-0096* and *SL-0119* have the same gene structure and are closely related. Therefore, the introns in each nTNL subfamily were gained independently (such as *SL-0062*) or from the common ancestor of a few closely related subfamilies (such as the common ancestor of the aforementioned six subfamilies).

In contrast, the intron features in different subfamilies of the TNL group are highly conserved. The pattern in Fig 1C indicates that the common ancestor of the TNL group might have 3 or 4 introns (as in the *N* gene). The first three introns of the *N* gene are conserved in most TNL subfamilies, with only a few exceptions. For example, the *Gro1-4* and the *SL-0159* subfamilies might have lost intron 2. The last intron (as in the *N* gene) in the TNL subfamilies might have been subjected to frequent gain and loss since it is present in some but absent in other subfamilies.

### An Integrated *R* Gene Map for Solanaceae Species Showing Prevalent P/A Polymorphism of *R* Gene Loci

**Fig 2:**
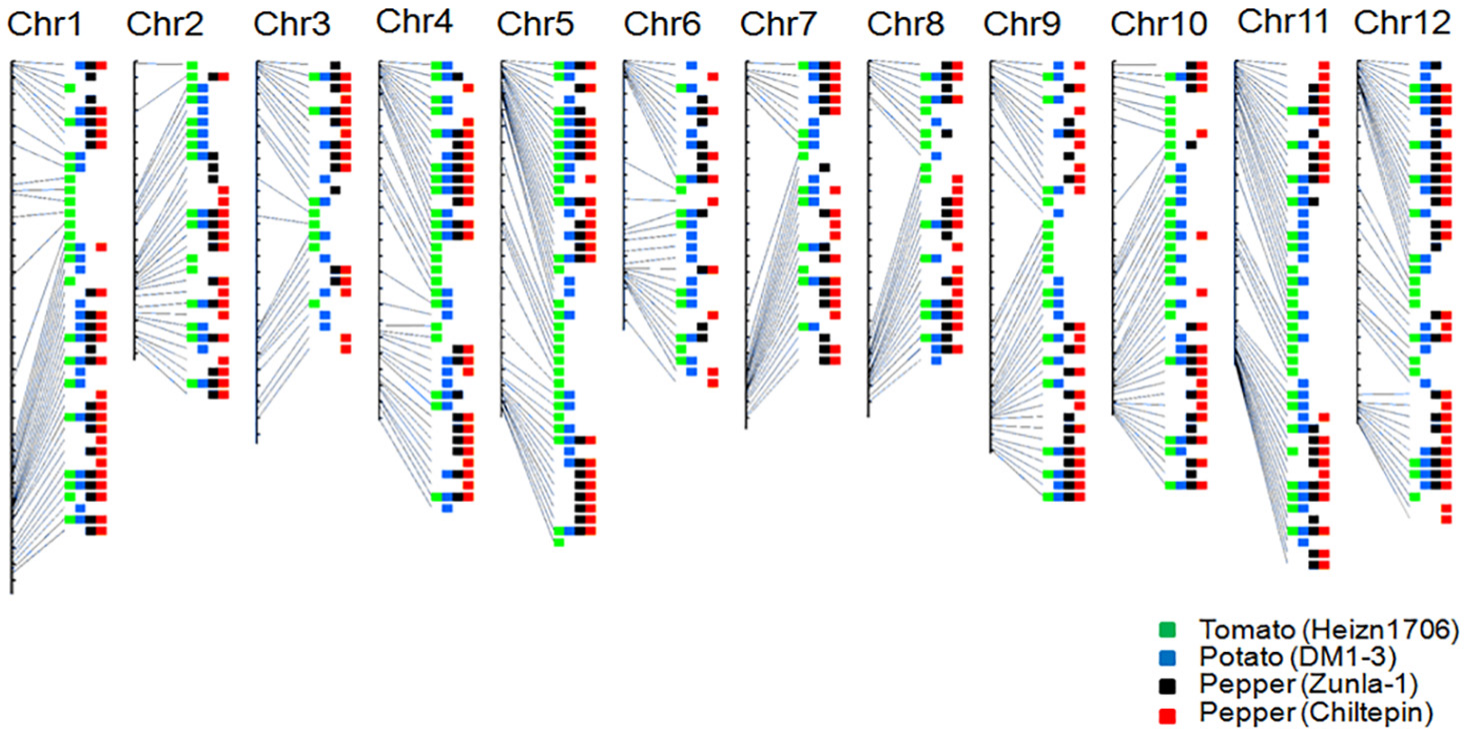
An integrated map of *R* loci in Solanaceae. *R* genes from different Solanaceae species were mapped onto the 12 chromosomes of tomato Heinz1706. *R* loci from tomato Heinz1706, potato DM1-3, pepper Zunla-1 and pepper Chiltepin are in green, blue, black and red, respectively.

*R* genes separated by no more than eight non-*R* genes are considered to be located at the same *R* gene locus (a multiple-copy locus) [5,33]. A total of 218, 298, 347 and 384 *R* gene loci were identified in tomato Heinz1706, potato DM1-3, pepper Zunla-1 and pepper Chiltepin (S5 Table). Of them, 238, 265 and 294 *R* loci from potato DM1-3, pepper Zunla-1 and pepper Chiltepin, which contain 761, 611 and 630 *R* homologs, respectively, were mapped into the syntenic regions of tomato chromosomes, while the remaining *R* genes failed to be mapped due to lack of enough flanking sequences. Consequently, an integrated map with 427 *R* gene loci was constructed (Fig 2). Of them, 371 *R* loci exhibit P/A polymorphism among Solanaceae species, while the other 56 *R* loci are shared by all four species (S2 Table). There are 176 loci specific to one individual species: 66 specific to tomato, 43 specific to potato, 67 specific to pepper (Zunla-1 and Chiltepin). This integrated *R* gene map will be helpful for future cloning of *R* genes from Solanaceae species.

### Copy Number Variation in Solanaceae Accounted for by a Few *R* Subfamilies

Of the 87 *R* subfamilies in Solanaceae, only 81, 79, 83, 80, 83 and 54 subfamilies were found in the genomes of tomato Heinz1706, tomato LA716, potato DM1-3, pepper Zunla-1, pepper Chiltepin and tobacco TN90, respectively (Table 2). Three subfamilies (*R1*, *DM-0233* and *DM-0846*) are present in potato but absent in tomato (Fig 1 and S6 Table). Similarly, six subfamilies (*R1*, *DM-0233*, *DM-0846*, *ZL-0810*, *ZL-1520* and *CH-0768*) were found in pepper but lost in tomato. The subfamilies with P/A polymorphism among different species are usually small and it does not account for the dramatic variation of *R* gene copy number among different species (S6 Table). In other words, the number of subfamilies in a genome is not the main cause of *R* gene number variations among different species. On the contrary, the average size of *R* gene subfamilies varies dramatically in a genome. The average number of *R* genes in an *R* subfamily in pepper (20.1 and 23.5 in Zunla-1 and Chiltepin, respectively) were at least three times as many as that in tomato (5.8 and 6.0) and tobacco (6.4) (Table 2).

The five largest subfamilies (*Rpi-blb2*, *BS2*, *SL-0273*, *Sw5-c* and *I2*) in pepper Zunla-1 and Chiltepin contain 832 (50.0%) and 1,027 (50.3%) *R* genes, respectively (S6 Table). In comparison, these five subfamilies have only 87 (18.2%), 95 (19.6%) and 73 (19.5%) *R* genes in tomato Heinz1706, tomato LA716 and tobacco TN90, respectively. The 22 largest *R* subfamilies contain approximately 80% of all *R* genes in pepper Zunla-1 (1,339) and Chiltepin (1,643). Therefore, the large variation of *R* gene number among Solanaceae species is mainly accounted for by a few *R* subfamilies.

### Copy Number Variation of the *Rpi-blb2* Subfamily

As the largest *R* subfamily in pepper, 298 and 366 *Rpi-blb2* homologs were found in pepper Zunla-1 and Chiltepin (S6 Table), respectively. Interestingly, the *Rpi-blb2* subfamily is Solanaceae specific, since no homologs were detected in Brassicaceae, Poaceae or Cucurbitaceae (Fig 1B). Of the chromosome-anchored *Rpi-blb2* homologs, the majority of them (> 78.0%) were located on chromosomes 5 and 6 of Solanaceae (S2 Table). Five *Rpi-blb2* loci in pepper genome have more than 10 copies. Consistent with above conclusion, the tomato Heinz1706 (28) and potato DM1-3 (86) genomes have relatively few *Rpi-blb2* genes (S6 Table). Nevertheless, the *Rpi-blb2* subfamily is the fourth and third largest subfamily in tomato Heinz1706 and potato DM1-3.

To analyze the evolution of the *Rpi-blb2* subfamily in Solanaceae, the nucleotide sequences (excluding short ones) of NBS-encoding region of 154 *Rpi-blb2* homologs (17, 29, 91 and 17 from tomato Heinz1706, potato DM1-3, pepper Zunla-1 and tobacco TN90, respectively) were aligned and a distance tree was constructed (S1 Fig). Two clades (I and II) are found on the tree and each clade has genes from all species included in this study, indicating two ancient lineages in the progenitor of Solanaceae. Many of the *Rpi-blb2* homologs of an individual species are grouped together, suggesting duplications after speciation. The majority (80%) of pairwise nucleotide identities of *Rpi-blb2* homologs of pepper in Clade I and II were lower than 90.3% and 90.5%, respectively, and there are only a few recent duplications (with nearly identical sequences). When *Rpi-blb2* homologs from all species (including wild tomato LA716 and wild pepper Chiltepin), the topology of the distance tree remains unchanged (data not shown). Ten pairs of obvious orthologs are found between tomato cultivar Heinz1706 and wild tomato LA716, and 125 pairs of orthologs between pepper cultivar Zunla-1 and wild pepper Chiltepin using bi-directional BLASTN. Sequence analysis of 59 nearly full-length (~3.5 Kb) *Rpi-blb2* homologs from pepper (both genotypes) found only 8 sequence exchanges (P < 0.05), consistent with independent evolution of *Rpi-blb2* homologs (data not shown).

### Copy Number Variation of the *BS2* Subfamily

The *BS2* subfamily showed dramatic number variation between Solanum (7 and 3 homologs in tomato Heinz1706 and potato DM1-3, respectively) and Capsicum (271 in pepper Zunla-1 and 355 in pepper Chiltepin) (S6 Table), consistent with previous report [15]. In Solanum, the ten *BS2* homologs from tomato Heinz1706 and potato DM1-3 were located at seven *R* loci, randomly distributed on five chromosomes (Chr02, 04, 08, 11 and 12) (S2 Table). Unfortunately, a large number of the *BS2* homologs in pepper (135 in Zunla-1 and 243 in Chiltepin) were not anchored onto chromosomes. Nevertheless, the majority of the chromosome-anchored *BS2* homologs in pepper (114 of 136 in Zunla-1 and 87 of 112 in Chiltepin) were mapped on chromosomes 7 and 9. Some *R* loci on chromosome 7 and 9 of pepper were species-specific and harbored dozens of *BS2* homologs. For example, the *R* loci ZL-locus-169 and -215 are specific to pepper Zunla-1, which contain 18 and 49 *BS2* homologs, respectively; the *R* loci CH-locus-187 and -244 are specific to pepper Chiltepin and contain 11 and 47 *BS2* homologs. Therefore, unlike most other species specific *R* loci (see above), the *BS2* homologs in pepper-specific *R* loci are large and contribute to the number variation of this subfamily between Solanum and Capsicum.

**Fig 3:**
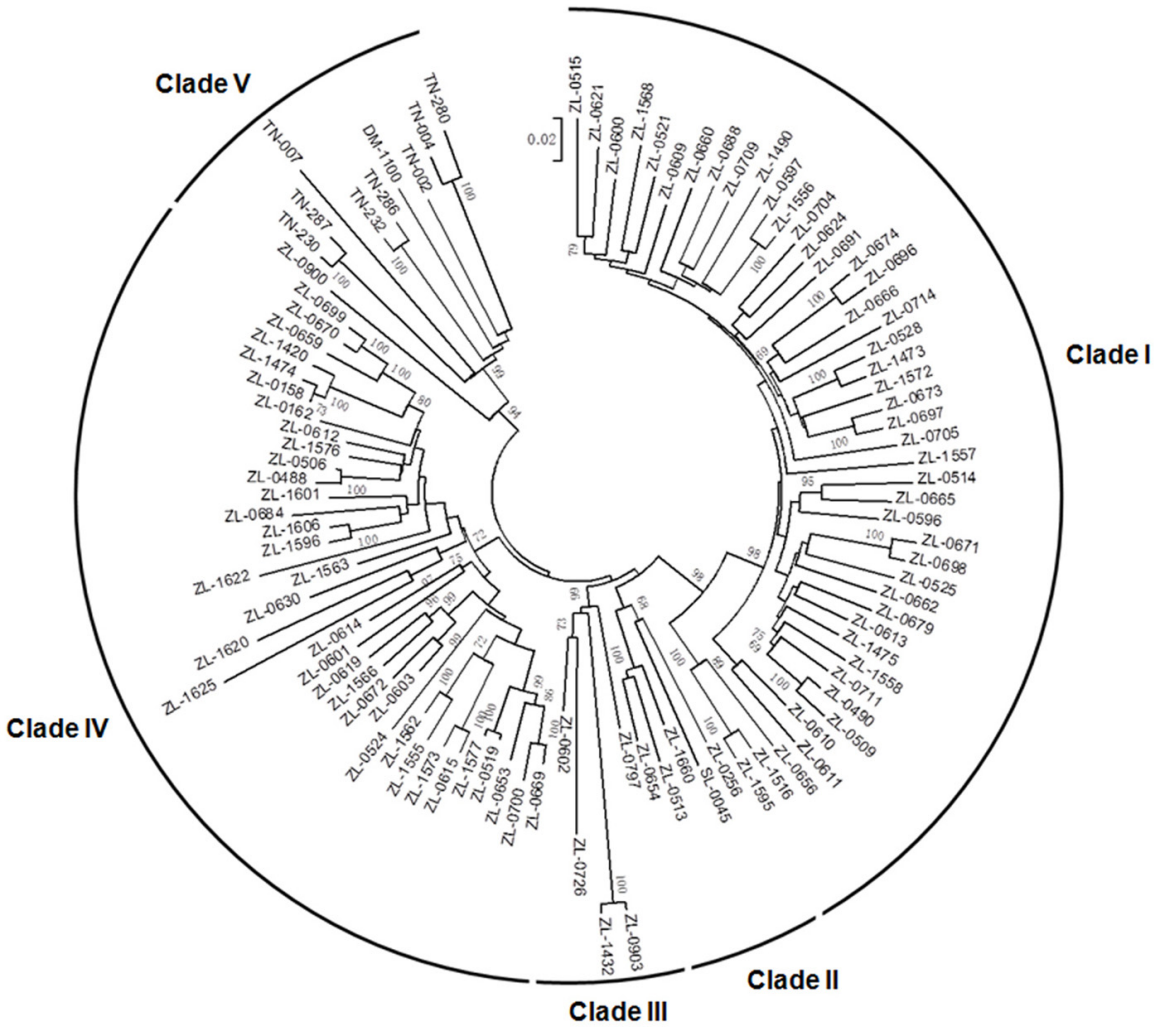
Distance tree of *BS2* homologs from Solanaceae species. Five clades can be found in the tree. Numbers on nodes are bootstrap values, and values <65 are not shown. Genes with name “*SL-*” are from tomato Heinz1706; genes with name “*DM-*” are from potato DM1-3; genes with name “*ZL-*” are from pepper Zunla-1; genes with name “*TN-*” are from tobacco TN90.

To understand the relationship of the *BS2* subfamily, a distance tree was constructed using nucleotide sequences (excluding short sequences) of the NBS-encoding region of 101 homologs from tomato Heinz1706 (1), potato DM1-3 (1), pepper Zunla-1 (91) and tobacco TN90 (8) (Fig 3). The only full-length *BS2* homologs from tomato and potato belong to two different clades, clade II and V, respectively. To better understand the evolution of the *BS2* subfamily, its expansion in pepper in particular, the eight partial *BS2* homologs from tomato (6) and potato (2), which were not used in constructing of the distance tree, were chosen to compare with all pepper *BS2* homologs in Fig 3. BLAST analysis showed that the eight partial *BS2* homologs belong to clades I, II and IV, respectively (S7 Table). Therefore, *BS2* homologs from Solanum are present in at least four of the five clades, while clade III is specific to pepper. The tree indicates that clades I-IV might have been present in the common ancestor of Capsicum and Solanum. After speciation, clade V was lost in tomato, while clade I and IV were lost in potato. In contrast, these four clades were maintained in pepper genome. Clades I and IV contain dozens of *BS2* homologs from pepper Zunla-1, obviously duplicated after speciation (Fig 3). The duplications of *BS2* homologs of clades I and IV after speciation predominantly accounted for the high copy number of the *BS2* subfamily in pepper.

To understand their evolution after duplication, sequence exchanges among 130 nearly full-length (~2.5 Kb) *BS2* homologs from pepper (both genotypes) were investigated. Only eight pairs of sequence exchanges were detected among 130 homologs (P < 0.05), suggesting independent evolution of most *BS2* homologs. A total of 145 pairs of orthologs were detected in all *BS2* homologs between pepper cultivar Zunla-1 and wild pepper Chiltepin (data not shown), consistent with the conclusion of independent evolution.

## Discussion

### The Current Method May Identify Additional *R* Genes from a Genome

*R* genes have been identified and analyzed from several plant genomes including Solanaceae species [3,5,8,10–15,17]. In previous studies, a species-specific NBS domain HMM profile was first constructed using hmmbuild implemented in the HMMER software. Then, the species-specific HMM profile was used to identify NBS genes in a genome with hmmsearch. Using above method, 54, 159, 435, 239, 1,015 and 327 *R* genes were detected in the genomes of papaya, Arabidopsis, potato, Brachypodium, apple and cassava, respectively, and these identified *R* genes all contain the NBS encoding sequences [9,10,13,16,23,34]. Obviously, partial *R* genes lacking NBS encoding sequences were missed in these studies. A new approach, the *R* gene enrichment and sequencing (RenSeq) workflow, successfully increased the numbers of *R* genes from 356 and 438 to 394 and 755 in the genomes of tomato and potato, respectively [14]. However, some *R* genes, such as partial ones, would not be detected using either of above methods. We have developed a greatly improved strategy for *R* gene identification from a sequenced genome, which was successfully used to identify hundreds of additional *R* gene homologs from a genome [5]. First, a species-specific R protein database was constructed and representative sequences were used as queries to do tBLASTn, and consequently intact or partial *R* genes (including genes lacking NBS domain) would be detected from a genome. Using this method, hundreds of additional *R* genes were identified from different genomes in this study (Table 1 and S1 Table). Though most of these newly identified *R* genes are partial genes, their alleles/orthologues may be full-length and functional, and therefore their identification may provide useful reference for future studies on *R* genes in these species.

### Variation of *R* Gene Copy Number among Different Species

The number of *R* genes from pepper Zunla-1 (1,665) and Chiltepin (2,042) is the largest in diploid genomes sequenced so far, which is similar to the number of *R* genes in hexaploid wheat [35]. Of the dozens of plant genomes sequenced so far, some genomes have small number of *R* genes, such as cucumber (70) and papaya (54) [16,17]. It remains unclear why the number of *R* genes can vary up to 40 times between different plant species. Theoretically, the more *R* gene homologs a genome has, the more functional resistance genes the species may harbor. However, the expansion of *R* gene homologs has fitness cost [36], and for each species, there should be a balance between resistance and fitness cost provided by all *R* gene homologs in a genome. Further studies are required to address the difference of such balance for different species.

The striking variation of *R* gene copy number may or may not exist within a plant family. The number of *R* genes from the Cucurbiteceae family is unexceptionally low [17]. On the other hand, the number of *R* genes may vary dramatically within a plant family. For example, the pepper genome (Chiltepin) has more than four times as many *R* genes as that in the tomato genome (Heinz1706) (Table 1 and S1 Table).

### The Majority of *R* Genes in Plants May Be Classified into the 87 *R* Subfamilies

In this study, 87 *R* subfamilies were classified in Solanaceae species, one gene (ref-gene) from each subfamily were annotated fully. Among these 87 ref-genes (including 73 well annotated and 14 functional *R* genes), 71 were nTNL genes and 16 were TNL genes (Table 2). Except partial ones, the remaining *R* genes identified from tomato Heinz1706 (97.9%), tomato LA716 (98.1%), potato DM1-3 (97.7%), pepper Zunla-1 (96.3%), pepper Chiltepin (95.4%) and tobacco TN90 (92.5%) were categorized into 81, 79, 83, 80, 83 and 54 of the 87 *R* subfamilies (Table 2). For comparative analysis in this study, 1,934, 159 and 147 *R* genes identified from Poaceae (rice, maize, sorghum and brachypodium), Brassicaceae (Arabidopsis) and Cucurbitaceae species (cucumber, melon and watermelon) were objected to compare with the 87 ref-genes using TBLASTX method [5,9,17]. As a result, 1,645 (85.1%), 159 (100%) and 146 (99.3%) of *R* genes in Poaceae, Brassicaceae and Cucurbiteceae were classified to one of the 87 Solanaceae *R* subfamilies (data not shown). In other words, the 87 *R* subfamilies contain the majority (> 85%) of *R* genes in the four plant families. In future studies, it will be very useful to identify additional *R* gene subfamilies from other plant species and construct a database with comprehensive *R* gene subfamilies, which represent all ancient *R* gene lineages. A comprehensive database of *R* gene subfamilies will facilitate studies on annotation, cloning and evolution of *R* genes in future studies.

### Extensive Amplification of a Few *R* Subfamilies May Increase *R* gene Copy Number Considerably

Although the number of *R* genes in pepper Zunla-1 and Chiltepin is considerably more than that in other Solanaceae species, the number of *R* subfamilies in different Solanaceae species is similar (Tables 1 and 2). Further analysis showed that subfamilies with P/A polymorphism between species are usually small and make little contribution to the copy number variation of *R* genes among species. More than 80% of all *R* genes were contributed by the top 24, 23, 20, 22, 22 and 27 largest *R* subfamilies in tomato Heinz1796, tomato LA716, potato DM1-3, pepper Zunla-1, pepper Chiltepin and tobacco TN90, respectively (S6 Table). As expected, functional *R* genes are more likely from the large subfamilies: 12 of the 14 cloned *R* genes from Solanaceae belong to these top largest *R* subfamilies. For instance, subfamilies *I2* and *Hero*, which harbor functional genes conferring resistance to *Fusarium oxysporum* f sp *lycopersici* and *Globodera rostochiensis*, respectively [37,38], are the third and ninth largest one in tomato; subfamilies *RB* and *Rpi-blb3*, which provide resistance to *Phytophthora infestans* [39,40], are the second and tenth largest one in potato, respectively; similarly, subfamily *N* is the fifth largest one in tobacco, conferring resistance to tobacco mosaic virus (TMV) [41]. The copy number of a subfamily may also vary dramatically. For example, subfamily *BS2* and *Rpi-blb2*, as the top two largest *R* subfamilies in pepper, have a total of 569 homologs in Zunla-1 and 721 in Chiltepin but they have no more than 90 homologs in Solanum and tobacco, respectively. The top five largest *R* subfamilies in pepper genomes contain nearly half of all *R* genes (832 of 1,665 and 1,027 of 2,042 in Zunla-1 and Chiltepin, respectively), while these five subfamilies harbor only 87 (18.2%), 95 (19.6%) and 73 (19.5%) *R* genes in tomato Heinz1706, tomato LA716 and tobacco TN90, respectively. In summary, the expansion of a few *R* gene subfamilies may substantially increase the copy number of *R* genes in a genome.

## Materials and Methods

### Identification of *R* Genes in Solanaceae Species

The genome data and gene models of tomato cultivar Heinz1706 (version 2.50), *S*. *pennelli* LA716 (version 1.0), potato DM1-3 (version 4.03), cultivar pepper Zunla-1 (version 2.0), wild pepper Chiltepin (version 2.0) and tobacco TN90 (SRP029183) were downloaded from corresponding web sites [26,27,29,30,32].

To genome-wide identify *R* genes in genomes, both Hidden Markov Model (HMM) and BLAST methods were used in this study [5]. Firstly, amino acid sequences of each genome were used to search against the HMM profile of NB-ARC domain (Pfam PF00931) using software HMMER3.0 with default parameter settings. Secondly, using key words, such as “NBS-LRR, NB ARC”, “ATP binding cassette” and “LRR kinase”, related protein sequences were retrieved from NCBI and a validating database was constructed, containing 3,146 NBS-LRR proteins, 3,098 ABC-transporter proteins and 6,758 kinase proteins. Then, the amino acid sequences identified from HMM searching were used as queries to search against the validating database, using BLASTP program with the E-value setting to 1e^-10^. Sequences with best hit of NBS-LRR or NB-ARC protein were used as R protein seeds to identify partial or intact *R* gene homologs from a genome using TBLASTN method (E-value cutoff of 1e^-10^). Finally, in order to confirm the results, following the rationale described above, all *R* homologs were validated again using non-redundant protein database from NCBI with BLASTX method (E-value cutoff of 1e^-10^). Once again, only sequences with best hit of NBS-LRR protein were considered as candidate *R* genes and used in further study.

Candidate *R* genes identified from tomato (Heinz1706), wild tomato (LA716), potato (DM1-3), pepper (Zunla-1 and Chiltepin) and tobacco (TN90) were named as *HZ-*, *PN-*, *DM-*, *ZL-*, *CH-* and *TN-* followed by a number, respectively.

### Annotation and Classification of *R* Genes

The following workflow was used to classify *R* genes into subfamilies. To simplify the calculations, all *R* genes of tomato Heinz1706 were first divided into subfamilies using BLASTN method with E-value setting to 1e^-10^. If a subfamily has a close homolog that has been well characterized in previous studies, the well characterized *R* gene was used as the reference gene and its gene model as the reference gene model for this *R* subfamily. In cases of subfamily with no well known *R* genes, full-length genes from the subfamily were chosen for manual annotation, including their intron/exon structure and intron phase. Gene model for full-length genes from the same subfamily are unexceptionally conserved. One of the manually annotated full-length genes was randomly chosen as the reference gene (ref-gene). Then, all *R* genes from other five Solanaceae genomes were compared with the ref-genes and classified to a subfamily using BLASTN method with E-value setting to 1e^-10^. *R* genes from the five genomes that have no similarity with any *R* gene subfamilies in tomato, were used to repeat above process: new subfamily classification and ref-gene annotation. If a *R* gene in a subfamily has different gene structure (large missing sequences or premature stop codon), it was considered as a partial gene or/and pseudogene.

Pfam and COILS were used to investigate if an R protein has TIR or CC domain, respectively [42,43]. All TNL and nTNL encoding genes were checked for intron position and intron phase. Three different intron phases in spliceosomal introns are defined: phase-0 as intron located before the first nucleotide of a codon, phase-1 as intron located before the second nucleotide of a codon and phase-2 before the third nucleotide of a codon [8,44,45]. The exon number, exon length and intron phase of ref-genes were annotated manually [5,17].

### Phylogenetic Analysis of *R* Genes

The coding sequences of *R* genes were translated into amino acid with a web server AUGUSTUS [46]. All the nucleotide (when all homologs from the same subfamily) or protein sequences (when homologs from different subfamilies) were aligned using program Muscle [47] and edited in GeneDoc. Mega 5.0 was used to construct Neighbor-joining (NJ) tree with Kimura two-parameter substitution model for nucleotide sequences and p-distance model for amino acid sequences [48]. Bootstrap value was calculated using 1,000 replicates, and a claimed clade mostly has a bootstrap value higher than 65. Sequence exchanges were detected using software Geneconv [49] with default parameters and confirmed manually.

### P/A Polymorphsim and An Integrated Map of *R* Genes

An *R* gene locus is defined as a locus with two or more *R* genes separated by no more than eight non-*R* genes [5,33]. If one or several *R* genes are present at a locus in a genome but none in another genome at the syntenic region, this locus is considered as P/A polymorphic locus [5]. All *R* gene loci were mapped onto tomato chromosomes according to the synteny of their flanking regions, resulting in an integrated map for Solanaceae [5].

## Supporting Information

S1 Data A fasta-file for all *R* gene sequences identified from tomato and potato genomes. (TXT)

S2 Data A fasta-file for all *R* gene sequences identified from pepper and tobacco genomes. (TXT)

S1 Fig Distance tree of *Rpi-blb2* homologs from Solanaceae species. Two clades, I and II, are in the tree. Numbers on nodes are bootstrap values, and values <65 are not shown. Genes with name “*SL-*” are from tomato Heinz1706; genes with name “*DM-*” are from potato DM1-3; genes with name “*ZL-*” are from pepper Zunla-1; genes with name “*TN-*” are from tobacco TN90.
(TIF)

S1 Table Details of *R* gene identified with our method compared with that reported in Jupe et al. (2013). (XLSX)

S2 Table Information of all *R* genes identified from Solanaceae species in this study. (XLSX)

S3 Table *R* gene numbers in some plants. (XLSX)

S4 Table Annotation of 87 reference genes in Solanaceae. (XLSX)

S5 Table Organization of *R* genes in Solanaceae species. (XLSX)

S6 Table Copy number of homologs in each subfamily in Solanaceae species. (XLSX)

S7 Table Information of best hits of eight partial *BS2* homologs from Solanum. (XLSX)
