# Supplementary figures and images for: Dramatic Number Variation of R Genes in Solanaceae Species Accounted for by a Few R Gene Subfamilies
Source: PLoS One. 2016 Feb 5;11(2):e0148708. doi: 10.1371/journal.pone.0148708 (PMC4743996; doi:10.1371/journal.pone.0148708)

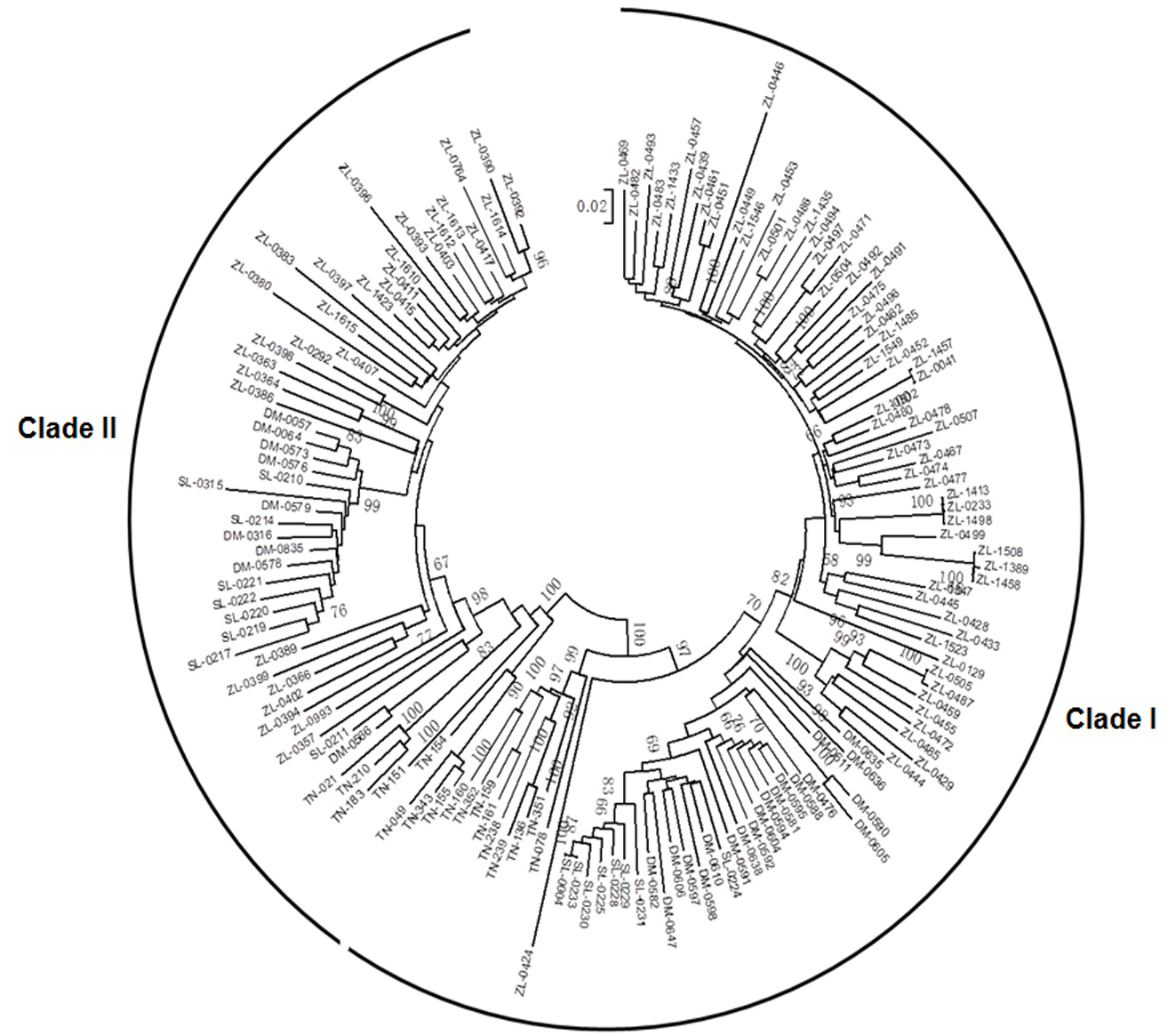

Supplement: S1 Fig — Two clades, I and II, are in the tree. Numbers on nodes are bootstrap values, and values <65 are not shown. Genes with name “SL-” are from tomato Heinz1706; genes with name “DM-” are from potato DM1-3; genes with name “ZL-” are from pepper Zunla-1; genes with name “TN-” are from tobacco TN90. (TIF) [file pone.0148708.s003.tif]
